# Potential of Fermentation and Vacuum Packaging Followed by Chilling to Preserve Black Soldier Fly Larvae (*Hermetia illucens*)

**DOI:** 10.3390/insects12080714

**Published:** 2021-08-10

**Authors:** Leen Van Campenhout, Dario Lachi, Dries Vandeweyer

**Affiliations:** 1 Research Group for Insect Production and Processing, Department of Microbial and Molecular Systems (M²S), Geel Campus, KU Leuven, 2440 Geel, Belgium; dario.lachi@kuleuven.be (D.L.); dries.vandeweyer@kuleuven.be (D.V.); 2 Leuven Food Science and Nutrition Research Centre (LFoRCe), KU Leuven, 3001 Leuven, Belgium

**Keywords:** black soldier fly larvae, *Hermetia illucens*, storage, fermentation, vacuum packaging, chilling

## Abstract

**Simple Summary:**

Insects are being produced at an industrial scale, mainly as feed ingredient to replace less sustainable protein sources in feed. Larvae of the black soldier fly (*Hermetia illucens*) are currently the most important species reared for this purpose. After production, it is necessary that the larvae be stored and transported in a stable way, i.e., without deterioration. In this study, we investigated fermentation and vacuum packaging technology as potential stabilisation techniques. Fermentation appears to be possible when the larvae are first blanched and pulverised, but bacterial endospores remain present and can potentially be dangerous if the conditions are not acidic enough. Vacuum packaging was tested as storage technique for living larvae, but their survival was lower than for living larvae packaged in air. Additionally, for killed larvae, vacuum packaging before chilling did not bring benefits over chilled storage alone. That was concluded from the fact that microbial counts were similar for larvae that were packaged in air or under vacuum during storage.

**Abstract:**

Black soldier fly larvae (*Hermetia illucens*) are currently reared at an industrial scale, mainly as a feed ingredient. The logistic chain not only involves the production of larvae, but also stabilisation, storage, and transport. The aim of this work was to study fermentation and vacuum packaging of larvae as potential preservation technologies. For fermentation, blanched larvae were pulverised into a paste, and a starter culture, NaCl, and glucose were added. The mixture was fermented for 7 days at 35 °C and then stored for 14 days at 4 °C and pH and microbial counts were monitored. Vacuum packaging was applied to living, blanched and frozen larvae. After packaging, they were stored for 6–10 days at several temperatures and gas composition, survival (living larvae) and microbial counts (killed larvae) were recorded. Fermentation allows storage of pulverised larvae, but points to consider are a rapid pH reduction and the presence of bacterial endospores. Vacuum packaging did not bring added value over cooling alone. This was the case for all types of larvae investigated. Vacuum packaging is not considered as a valuable preservation technology to pursue for storage and transport of black soldier fly larvae.

## 1. Introduction

The volume of edible insects produced has increased over the last few years and is expected to increase further [1,2]. According to a factsheet [3] of the International Platform of Insects for Food and Feed (IPIFF), in 2019, European insect producers delivered 500 tonnes of whole insects, insect ingredients and insect-containing finished products. The growth of the market is forecasted by IPIFF to reach 260,000 tonnes by 2030. Likewise, a recent report by Rabobank predicts an increase of the demand for insect protein to 500,000 tonnes as feed ingredient in the same time span [4]. An increase in production volumes requires the whole logistic chain, including storage and transport steps, to be upscaled in parallel. The opportunity to produce more and larger batches of insects comes with the need for knowledge on possible methods to stabilise insects, as intermediate or finished product, and preferably including a cost-benefit analysis. Thus far, dehydration has received most attention both in industry, as well as in research as preservation technology for insects. The removal of water from a product is known to retard or even stop chemical and microbiological deterioration processes [5]. Several studies have focused on chemical and/or microbiological stability during storage of dried edible insects. For example, Larouche et al. [6] and Kamau et al. [7,8] studied these aspects for larvae of the black soldier fly (*Hermetia illucens*), Kinyuru et al. [9] and Fombong et al. [10] for the longhorned grasshopper *Ruspolia differens*, and the former also for the winged termite *Macrotermes subhylamus*, Azzolini et al. [11], Vandeweyer et al. [12], Lenaerts et al. [13], Kroencke et al. [14,15] for the yellow mealworm (*Tenebrio molitor*), Lee et al. [16] for the cricket species *Gryllus bimaculatus*, Kamau et al. [7], Lucas-Gonzalez et al. [17] and Bawa et al. [18] for the cricket species *Acheta domesticus* and Vandeweyer et al. [19] for the cricket species *Gryllodes sigillatus*. Drying technologies included in the aforementioned studies involve mainly oven drying, freeze-drying, microwave drying and solar drying. Zhen et al. [20] focused on the production of meal from black soldier fly larvae (BSFL) and studied the impact of six killing methods followed by oven drying and grinding on the chemical composition and in vitro digestibility of the meal. Generally, as based on the aforementioned studies, when the moisture content and water activity can be reduced substantially, drying has proven to be a valuable technology to increase the shelf life of edible insects. However, it comes with a considerable investment and/or operational cost, certainly oven, freeze-drying and microwave drying. It is generally known that removing water from a matrix is a costly step in any feed or food processing chain.

The insect production sector is looking for alternative technologies, that do not involve the removal of water but can still increase the shelf life of produced insects based on other strategies. Indeed, not all applications of insects require them to be dried before further processing into the application. In animal feed, the currently largest application domain of mass reared insects, a small part of the feed ingredients in formulations are liquid ingredients. While in the current practice dried insects are processed in animal feed, the mixing of (eventually pulverised, but not dried) insects in a feed may be possible. Another application domain of mass-reared insects where drying is not a required step, is in the recovery of antimicrobial peptides (AMPs) from insects. Several insect species, and in particular the black soldier fly, are known to express a range of AMPs as part of their immune system. The knowledge on their composition, functions and potential applications in drug development increases rapidly [21,22]. This evokes questions in possible technologies for downstream processing and recovery of these interesting molecules after harvest of insects, in the case the molecules are produced on a large scale in the insect (and not by heterologous expression in a micro-organism, for instance).

This paper envisages two technologies that do not require the removal of moisture and that can potentially increase the shelf life of insects: fermentation and vacuum packaging. These processes were considered as potentially implementable at industrial scale as preservation technologies for BSFL. The work focuses on one insect species, i.e., BSFL. Fermentation is defined as “any process for the production of a product by the mass culture of microorganisms”, and improving the shelf life of the fermented biomass is one of the reasons why fermentation can be applied [23]. The improvement of the storage stability is based on acidification of the matrix, either by micro-organisms endogenously present in the matrix (spontaneous fermentation) or by added starter cultures, thereby generally reducing microbial growth. The reduction of the pH is accomplished by the microbial production of specific metabolites, organic acids, such as lactic acid, during fermentation. Other metabolites produced, such as hydrogen peroxide or bacteriocins, can assist in the growth inhibition of spoilage organisms and pathogens during fermentation and eventually storage after fermentation [24]. In our previous research, we demonstrated that yellow mealworms can be fermented successfully, when they are pulverised and when a starter culture is added [25,26,27]. The storage stability after fermentation was even better than when preservatives were added to the mealworm paste instead of fermentation [28]. Hence, the question came up whether that fermentation procedure would be successful for BSFL too.

The rationale to test the second technology on (whole) BSFL relates to the fact that Modified Atmosphere Packaging (MAP), which also encompasses vacuum packaging, is a commonly applied method in the food industry to preserve a wide range of foods [29]. In vacuum packaging, the product is transferred into a packaging material with high barrier properties for O_2_, CO_2_, N_2_ and H_2_O. The air is removed from the package by bringing it under vacuum, and then the package is sealed [29]. By removing the air, and hence the O_2_, oxidation as well as growth of aerobic micro-organisms is limited [30]. The technology could be adopted by the insect industry in a relatively easy way, since equipment exists for vacuum packaging of bulk particulate products (such as larvae). However, no information is available in the scientific literature on the potential of the technology. For foods, MAP is applied for non-respiring food products. In addition, fresh (and respiring) vegetables are packaged in reduced oxygen concentrations, in order to lower their respiration rate and in this way keep their fresh appeal. This brought the idea to test vacuum packaging not only on killed (non-respiring) BSFL, but also on living (respiring) larvae as a potential storage method.

**Figure 1:**
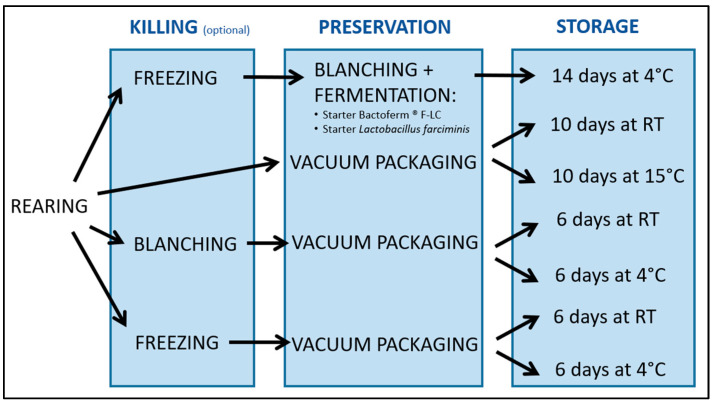
Overview of the experimental design (RT: room temperature).

The aim of this work was to investigate the effect of fermentation and of vacuum packaging followed by cooled storage on some physical properties, on survival in the case of living larvae and on microbial counts in the case of killed larvae. An overview of the experimental design is given in Figure 1. The results were used to assess the feasibility and the added value of the technologies for BSFL processing and storage.

## 2. Materials and Methods

### 2.1. Fermentation Followed by Storage and Sampling

Larvae for fermentation were obtained in a frozen state from two producers (Protix, Dongen, The Netherlands, and Inagro, Roeselare, Belgium). Two starter cultures, Bactoferm^®^ F-LC (Chr. Hansen, Pohlheim, Germany) or *Lactobacillus farciminis* (Chr. Hansen), proven to be successful in the fermentation of the yellow mealworm (*Tenebrio molitor*) [25,26,27] were used in separate experiments. For each starter, three consecutive fermentations were set up, one with larvae from Protix and two with larvae from Inagro. The fermentation process was carried out as described by Borremans et al. [27]. First, 300 g larvae were blanched for 40 s in 1 L boiling water and drained in a sieve. Then, larvae were pulverised with a sterilised hand-held mixer (Bosch CNHR 25), and 2.8% NaCl (*w*/*w*) and 0.75% D-glucose (*w*/*w*) were added. The BSFL paste was mixed again and four 50 mL Falcon tubes were each filled completely with paste to serve as control. The remaining paste was inoculated with a freeze-dried culture of one of the starters to a concentration of approximately 6.5 log cfu/g and homogenised. With this inoculated paste, four other Falcon tubes were filled completely. All tubes were closed firmly with a screw cap. Through the caps of one control and one inoculated tube, a pH electrode was inserted for on-line pH measurement. All caps were sealed with parafilm and incubated for 7 days at 35 °C (conditions based on the suppliers’ instructions of the starters and on preliminary experiments).

To perform microbial counts and off-line pH measurements, three samples were taken from both (control and inoculated) BSFL pastes before fermentation. After fermentation, all tubes without pH electrode were opened and a sample was taken. Then the tubes were closed and stored refrigerated (set point: 4 °C) for 14 days, after which they were sampled again.

### 2.2. Vacuum Packaging Followed by Storage and Sampling

Vacuum packaging followed by storage was investigated first as storage technique for living larvae with monitoring of gas composition and larval survival, and subsequently for killed larvae, with focus on gas composition and microbial counts.

For vacuum packing of living larvae, two replicate experiments were executed, one with larvae obtained from Inagro and one with larvae obtained from RADIUS (Thomas More Kempen, Geel, Belgium). All larvae were at the end of their rearing cycle. In each experiment, 40 portions of 200 g BSFL were weighed in high-barrier vacuum bags (Colamin multi-layer PA/EVOH/PA/PE, 200 × 250 mm, Euralpack, Schoten, Belgium). Of those bags, 20 were packed without vacuum (i.e., only sealing, to serve as control) and the other 20 by applying 35 mbar (absolute pressure) vacuum before sealing using a Multivac C200 packing machine (Wolfertschwenden, Germany). From each group, 10 bags were stored in a closed box at room temperature and 10 bags were stored in a cooled incubator (KB115, Binder, Tuttlingen, Germany) at 15 °C. During a storage period of 10 days, the gas composition of each package was monitored daily and each day survival was measured for one package per treatment as described below.

Vacuum packaging of killed larvae was performed on one batch of larvae obtained from Inagro. It was hypothesised that the killing technique can affect the microbial load after killing, and hence the microbial dynamics during storage. Therefore, one part of the larvae was killed by blanching (40 s in boiling water followed by 1 min cooling in cold water) and the other part by freezing (16 h at −20 °C followed by 6 h thawing at 4 °C). After killing, 8 portions of 200 g larvae from each killing method were packed in high barrier bags, without (control, 4 bags) or with (4 bags) vacuum and stored at room temperature or at 4 °C (see above). Bags were stored for 6 days. Gas composition was measured daily in each bag. Microbial counts were determined for one bag of each condition, prior to packing and at day 6.

### 2.3. Determination of pH during Fermentation and Storage

To follow up the pH during the fermentation and the subsequent refrigerated storage, both on-line and off-line measurements were executed. Off-line pH (individual measurements) was determined in the BSFL paste before fermentation and again when opening each Falcon tube after fermentation as well as after storage. A digital pH meter (Portamess 911, Knick, Berlin, Germany with SI analytics electrode, Mainz, Germany) was used for the off-line measurements. On-line pH (continuous measurements) was determined in one additional Falcon tube per condition (control and with starter culture) per experiment. To allow on-line monitoring, the electrode (SenTix 82, VWR, Leuven, Belgium) of a continuous pH meter (pHenomenal pH 1100H, VWR) was introduced in the middle of the tube through a hole in the screw cap and sealed with parafilm. On-line pH was monitored by automatically registering the pH every 15 min during the complete fermentation and storage period without opening the Falcon tube.

### 2.4. Determination of Gas Composition during Vacuum Packaging and Storage

During storage of the packaged BSFL (living or dead, with or without vacuum), the concentration of the gases O_2_ and CO_2_ was determined daily in each package’s headspace when possible (packages completely vacuum could not be measured). The first measurement was executed 30 min after sealing and the last one immediately before opening them for analysing survival or microbial counts. Since a destructive sampling plan was followed, removing one package per condition per sampling moment, the number of replicas for gas measurements was reduced every sampling moment until one sample was left. All gas measurements were performed using a CheckPoint II O_2_/CO_2_ m (PBI Dansensor, Ringsted, Denmark) by puncturing the package with a 0.5 mm needle through a single-use septum (PBI Dansensor) applied on the package prior to each measurement.

### 2.5. Determination of Survival during Storage of Living Larvae

After packaging the living BSFL with or without vacuum, their survival during storage under the conditions applied was evaluated daily. To this end, larvae from one package per condition were first exposed to ambient air for at least one hour after opening the package to recover. Living larvae were then selected as the larvae that were still vivid or mobile compared to completely immobile larvae that were considered dead. The survival was finally calculated from the mass of remaining living larvae compared to the total mass of larvae in the package.

### 2.6. Microbial Counts

Samples from the fermentation experiments and from the vacuum packaging experiments using killed BSFL before and during storage were subjected to microbial counts. According to the ISO standards compiled by Dijk et al. [31], the total viable aerobic count (TVC) and the amount of lactic acid bacteria (LAB), Enterobacteriaceae (ENT) and aerobic endospores (AS) were determined for each sample. First, whole larvae samples were pulverised with a flame-sterilised hand-held mixer (Bosch CNHR 25). Samples of (fermented) BSFL paste were used without this pre-treatment. Next, 5 g of each sample was added to 45 g peptone physiological salt solution (0.85% (*w*/*v*) NaCl; 0.1% (*w*/*v*) peptone (Biokar Diagnostics, Beauvais, France)). This primary dilution was homogenised (Bagmixer 400W, Interscience, Saint Nom, France) for 1 min in a stomacher bag. Ten-fold dilutions were then plated on Plate Count Agar (PCA, Biokar Diagnostics) and incubated for 72 h at 30 °C for the determination of TVC, on de Man, Rogosa and Sharpe agar (MRS, Biokar Diagnostics) and incubated 72 h at 30 °C for LAB, and on Violet Red Bile Glucose agar (VRBG, Biokar Diagnostics) and incubated for 24 h at 37 °C to determine ENT. For the enumeration of AS, the primary 10^−1^ dilution was first given a heat treatment for 10 min at 80 °C before plating a dedicated ten-fold dilution onto PCA and incubation for 48 h at 37 °C. Microbial counts were calculated as log colony-forming units (cfu) per gram.

### 2.7. Statistical Analyses

Statistical analyses were executed using JMP Pro^®®^ version 15.2.1 (SAS Institute Inc.; Cary, NC, USA). First, normal distribution fit was tested using the Shapiro-Wilk test. Differences in off-line pH measurements were determined by One-Way ANOVA followed by All Pairs Tukey–Kramer HSD post hoc test. In case of unequal variances (as determined by Levene’s test), Welch ANOVA followed by Steel-Dwass All Pairs post hoc test was used instead. For microbial counts, differences between sampling moments were determined using a Kruskal-Wallis test followed by Dunn All Pairs post hoc test. Additionally, differences per condition and per microbial count between inoculated and uninoculated samples were determined using Mann–Whitney–Wilcoxon tests. A significance level of 0.05 was adopted for all statistical tests.

## 3. Results

### 3.1. Fermentation Followed by Storage

#### 3.1.1. pH Reduction

**Figure 2:**
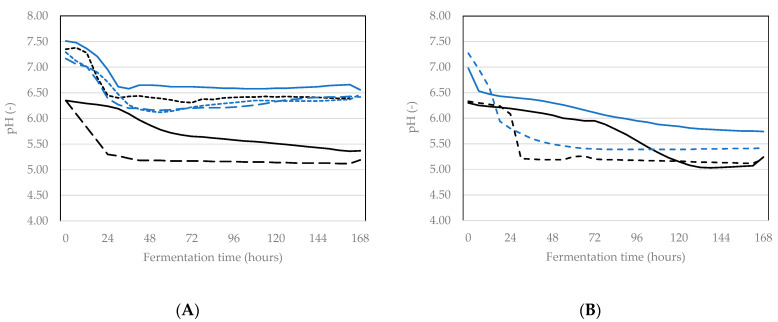
On-line pH measurements during incubation of control samples and samples inoculated with a starter culture (**A**) with starter Bactoferm^®^ F-LC (three replicates of control and starter were monitored on-line); (**B**) with starter *Lactobacillus farciminis* (two replicates of control and starter were monitored on-line). Control 1: black, full line, Control 2: black with broad dashes, Control 3: black with small dashes, Starter 1: blue, full line, Starter 2: blue with broad dashes, Starter 3: blue with small dashes.

When fermentation processes are developed for food or feed matrices, a key parameter to judge the process is pH. Obviously, besides pH other properties of the fermented food or feed need to also be evaluated to determine whether it is viable for fermentation, such as the nutritional profile and the taste of the product. However, pH is a parameter that can easily be determined when applying fermentation for the first time to a matrix and during further process optimisation. To assess the fermentation process, in our work, we determined the pH off-line before and after incubation as well as on-line in some of the samples. Before incubation, all BSFL pastes had a pH of close to 7.00 as determined by off-line measurement (Table 1), but the on-line measurements showed a larger range of values between 6.30 and 7.50 (Figure 2). For the off-line measurements, there were no statistical differences between control and starter in each of the two sets of experiments with a specific starter (*p* > 0.05). It can also be seen from Table 1 that the pH reduction during incubation was significant for both control and starter in the Bactoferm^®^ F-LC experiment, but not in the *L. farciminis* experiment. The off-line measurements revealed a difference in pH before and after incubation of 0.88, 1.03, 0.01, 0.79 for the treatment without and with starter in the Bactoferm^®^ F-LC experiment and the treatment without and with starter in the *L. farciminis* experiment, respectively. For the on-line measurements, these differences were 0.97, 0.84, 1.10 and 1.55. Therefore, except for the on-line measurements in the Bactoferm^®^ F-LC experiment, the data reveal a slightly larger pH reduction in samples where starter is used compared to samples without starter.

A question that comes up when developing a fermentation process for a certain matrix, is how long the fermentation should be conducted. The pH should not only drop to a value that is low enough (see also Discussion), but the reduction should also be rapid. The online measurements (Figure 2) disclose that, except for Control 1 in the experiments with both starters, the pH reduction is most pronounced within the first 24 h of fermentation. When Bactoferm^®^ F-LC was used, after 24 h no further reduction in pH was observed, while the reduction when using *L. farciminis* continued also after 24 h.

Off-line pH measurements were not only performed before and after fermentation, but also after 14 days storage at 4 °C. No significant differences occurred in pH for any of the treatments between the start and the end of the storage period.

#### 3.1.2. Microbial Counts

**Figure 3:**
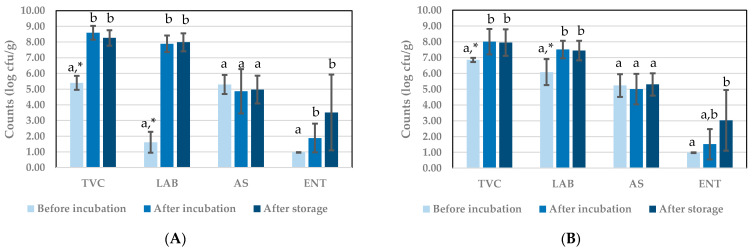
Microbial counts during experiments to investigate the potential of starter Bactoferm^®^ F-LC: (**A**) replicates incubated without starter (control); (**B**) replicates incubated with starter. Results are the mean of three replicates (*n* = 3). TVC: total viable counts; LAB: lactic acid bacteria counts; AS: aerobic endospore counts; ENT: Enterobacteriaceae counts. ^a,b^ Means per microbial count with the same superscript do not differ significantly. * Corresponding means indicated with asterisks are significantly different.

**Figure 4:**
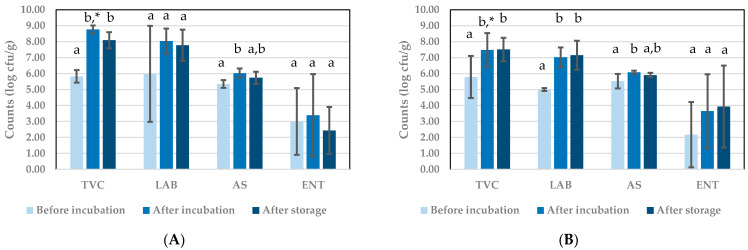
Microbial counts during experiments to investigate the potential of starter *Lactobacillus farciminis*: (**A**) replicates incubated without starter (control); (**B**) replicates incubated with starter. Results are the mean of three replicates (*n* = 3). TVC: total viable counts; LAB: lactic acid bacteria counts; AS: aerobic endospore counts; ENT: Enterobacteriaceae counts. ^a,b^ Means per microbial count with the same superscript do not differ significantly. * Corresponding means indicated with asterisks are significantly different.

Results obtained after performing plate counts of the BSFL paste before and after fermentation and after refrigerated storage of the fermented product are presented in Figure 3 for tests with starter Bactoferm^®^ F-LC and in Figure 4 for tests with *L. farciminis.* The TVC for the uninoculated paste was between 5.00 and 6.00 log cfu/g, which can be considered as relatively high, knowing that the larvae were blanched before processing into paste. This can be due to the presence of bacterial spores, as discussed further. Inoculation can be expected to raise the average TVC, but this was only observed in the series of experiments with Bactoferm^®^ F-LC. In all cases, TVC increased significantly with 1–3 log cycles during incubation, but then remained constant (no significant differences between TVC after incubation and after storage).

In experiments with starter Bactoferm^®^ F-LC (Figure 3), the LAB counts before incubation in uninoculated samples were low as a result of blanching, but they were multiple log cycles higher in inoculated samples, obviously due to the presence of the starter. Hence, both TVC and LAB before incubation were significantly different in Figure 3B compared to Figure 3A. In experiments with *L. farciminis* (Figure 4), a large variation was observed in LAB counts in uninoculated samples, indicating a variable effect of blanching on the presence of LAB in these samples. In all experiments, a clear growth of LAB during incubation was recorded with a significant difference between samples taken before and after incubation, for the uninoculated and inoculated samples in the experiments with Bactoferm^®^ F-LC and for the inoculated samples in the experiments with *L. farciminis*. BSFL paste can therefore be considered as a matrix which is well suited for LAB growth.

In all tests, the pastes contained considerable amounts of aerobic bacterial endospores at levels around 5.00 and 6.00 log cfu/g and the numbers remained constant during incubation and subsequent refrigerated storage. In the series of experiments with *L. farciminis*, statistically significant differences were found between the three time points were samples were taken, but from a microbiological point of view, a difference in counts is only meaningful when it is larger than 1 log cycle. Therefore, the AS counts can be considered as remaining constant throughout all experiments.

ENT counts were very low in the BSFL paste prepared for the Bactoferm^®^ F-LC experiments, but more variation on their initial counts was recorded in the *L. farciminis* experiments. Low counts can be expected after blanching of the larvae, because in general Enterobacteriaceae are heat-sensitive [32]. In the Bactoferm^®^ F-LC experiments, ENT counts increased during incubation and further during storage afterwards, yielding values that were significantly and about 2 log cycles higher in stored products compared to initial counts before fermentation. In the *L. farciminis* experiments, larger standard deviations were observed, preventing the observation of clear dynamics. Overall, after incubation and storage, ENT counts ranged between 2.43 and 3.93 log cfu/g.

### 3.2. Vacuum Packaging Followed by Storage

#### 3.2.1. Vacuum Packaging and Storage of Living Larvae

**Figure 5:**
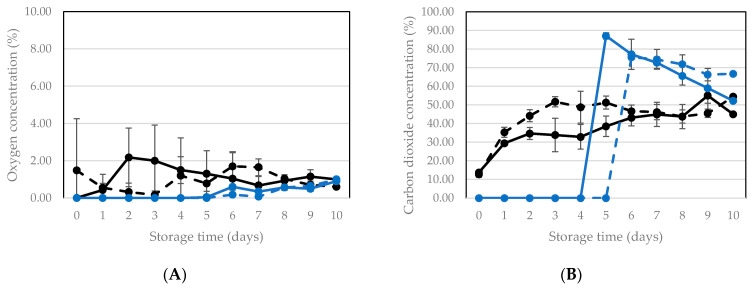
Oxygen (**A**) and carbon dioxide (**B**) concentration during storage at room temperature of packages of living larvae that were only sealed (no vacuum = control) and that were vacuum packaged prior to storage. At day 0 and 1, data points are the mean of 10 packages (*n* = 10). Afterwards, each day there was one replicate less, since one package was opened to determine survival. Vertical bars are standard deviations. Two experiments were performed with different batches of larvae: Control 1: black, full line, Control 2: black dashed line, Vacuum 1: blue, full line, Vacuum 2: blue dashed line.

**Figure 6:**
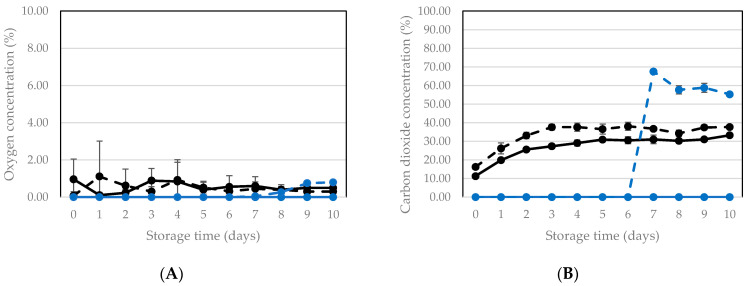
Oxygen (**A**) and carbon dioxide (**B**) concentration during storage at 15 °C of packages of living larvae that were only sealed (no vacuum = control) and that were vacuum packaged prior to storage. At day 0 and 1, data points are the mean of 10 packages (*n* = 10). Afterwards, each day there was one replicate less, since one package was opened to determine survival. Vertical bars are standard deviations. Two experiments were performed with different batches of larvae: Control 1: black, full line, Control 2: black dashed line, Vacuum 1: blue, full line, Vacuum 2: blue dashed line.

When assessing the potential of MAP as a preservation technology, typically the O_2_ and CO_2_ concentrations in the package during storage are monitored. When applying vacuum packaging, obviously the aim is to maintain the reduced headspace volume and consequently the low concentration of both gases as long as possible. The O_2_ and CO_2_ profiles in packages of living BSFL, stored at room temperature and 15 °C can be seen in Figure 5 and Figure 6, respectively. In control packages (only sealed but no vacuum), the O_2_ levels varied between 0.01% and 2.00%. The O_2_ concentration measured in these packages is the result of O_2_ entrance via the packaging material on the one side and the use of O_2_ by the larvae and aerobic micro-organisms. At higher temperatures, the permeability for O_2_ (and other gases) of the packaging materials used is higher [33], which can explain why the levels at room temperature on average were higher than at 15 °C. In vacuum packaged products, an increase in O_2_ in the package during storage can point to O_2_ entrance via the packaging material, or via (micro)leakages in seals and/or the packaging material. However, for preservation the aim is to maintain as low as possible O_2_ concentrations for as long as possible in order to exclude growth of aerobic micro-organisms. For both storage temperatures, the O_2_ concentration remained below 1.00% in vacuum packaged samples throughout the whole storage period of 10 days. In vacuum packaged samples stored at room temperature, oxygen was present throughout the whole experiment between 1 and 2%. In packages stored at 15 °C, no oxygen was present during the first five days and from then on, a low level around 1% was observed. This can be explained by the fact that, as mentioned earlier, at higher temperatures the permeability of the packaging materials used is higher.

An increase in CO_2_ in the package during storage can originate from CO_2_ production by the larvae and from microbial respiration. In control packages, a moderate yet clear increase was observed during the first three days, to reach equilibrium values between 40 and 50% at room temperature and between 30–40% at 15 °C. In the vacuum packaged samples stored at room temperature and in the second experiment with storage at 15 °C, a steep rise in CO_2_ occurred at 4–6 days. That increase was clearly more pronounced than in the control packages. As soon as the CO_2_ rapidly increases, the packages are no longer vacuum, and this coincides with the small increase in O_2_, as mentioned before, by permeation through the packaging material.

**Figure 7:**
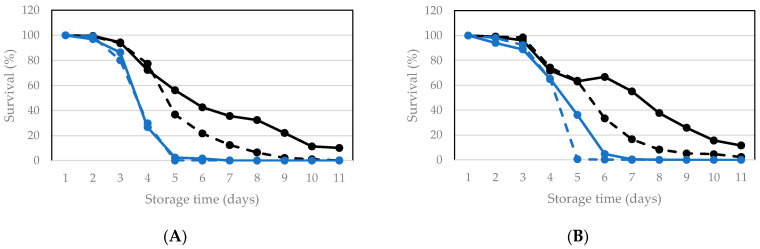
Survival of larvae during storage at room temperature (**A**) and at 15 °C (**B**) after being packaged and only sealed (no vacuum = control) and after being vacuum packaged. Each data point represents 1 package. Two experiments were performed with different batches of larvae: Control 1: black, full line, Control 2: black dashed line, Vacuum 1: blue, full line, Vacuum 2: blue dashed line.

Vacuum packaging was applied for living larvae and their survival was monitored during subsequent storage by daily determining the percentage of living larvae in one package (Figure 7). For the two storage temperatures, vacuum packaging caused survival to drop faster than only sealing. Taking the data obtained at the two temperatures together, the four control survival curves all decrease slower than the four survival curves for vacuum packaging. Hence, from survival point of view, it is better to simply seal packages than to remove the air and then seal them. When comparing the control packages for the two temperatures, the average survival (obtained by averaging values of control 1 and 2) declined somewhat faster at room temperature than at 15 °C (for instance, 46 and 63% average survival at day five at room temperature and 15 °C, respectively; 32 and 50% at day six; 24 and 36 at day 7). That difference diminishes further in the storage period. Therefore, a practical guideline can be formulated based on these data, that when living larvae need to be stored between 3–7 days, cooling to 15 °C is useful but vacuum packaging is not.

#### 3.2.2. Vacuum Packaging and Storage of Killed Larvae

When blanched larvae were packaged without applying vacuum and then stored at room temperature, the O_2_ concentration (Figure S1) decreased to zero in two days and remained zero, pointing towards consumption of all oxygen that was still present in the headspace after packaging. In parallel, the CO_2_ concentration increased to levels of about 35%, also demonstrating microbial respiration. Indeed, microbial numbers (Table 2) were low after blanching, but increased substantially during storage. Vacuum packages stored at room temperature remained vacuum and no O_2_ and CO_2_ were measured throughout the experiment. Nevertheless, microbial numbers also increased during storage. Values for the total viable count, lactic acid bacteria and endospores were somewhat lower than for storage without vacuum, but not in the way that the vacuum application provided a meaningful microbial reduction. When blanched and packaged larvae were stored at 4 °C, the situation was clearly better than for storage at room temperature. For control packages stored at 4 °C the gas composition remained exactly as it was immediately after packaging (i.e., the concentrations of air of about 21% O_2_ and CO_2_ below 1%), and vacuum packages no O_2_ and CO_2_ were measured throughout the experiment. Microbial numbers remained low throughout the experiment and the refrigerated storage had a clear impact on microbial reduction.

For larvae that were frozen, then packaged just by sealing (control) and stored at room temperature, the O_2_ in the headspace (Figure S2) was consumed rapidly and remained zero. Also, the CO_2_ concentration showed an enormous increase in two days to levels of about 60–70%. This can likely be explained by an intensive microbial activity. In contrast to killing by blanching, freezing did not result in low microbial numbers (Table 2) in the biomass at the start of the storage period. Numbers were already high when the larvae were packed. It can be seen from the gas profiles as well as from the microbial numbers that the application of vacuum could not reduce microbial activity nor counts. When frozen larvae, packaged without or with vacuum, were stored at 4 °C, the situation was better than for storage at room temperature. For control packages, the O_2_ concentration (Figure S2) showed an initial decrease but then increased slightly, indicating that not all O_2_ entering the package via the material was consumed by micro-organisms. In parallel, the CO_2_ concentration increased but not to the same level as in room temperature, and it even showed a decrease in the second half of the experiment, indicating that more CO_2_ left the package via the material than that it was produced by the microflora. Both for control and vacuum packages, the total viable counts and the counts of the lactic acid bacteria, a group of micro-organisms that contains several spoilage organisms, were more than 1 log cycle lower at the end of the storage than at the start. Hence, a fraction of the microbiota even died off during storage. However, when comparing vacuum to control, it cannot be observed that vacuum packaging implies an additional hurdle for the microbiota.

## 4. Discussion

Research on the fermentation of edible insects to improve their shelf life, and in particular of BSFL, is scarce. Mogodiniyai Kasmaei et al. [34] investigated a 98-days fermentation process of gently crushed larvae in 100 mL glass-tubes with water locks. They tested several additives during fermentation, but not starter cultures. They concluded that this approach was not suitable due to the high biomass loss (10–28%) during fermentation, the absence of a significant pH reduction (end values between 5.3 and 6.8) and the bad smell of the end product. Kube et al. [35] investigated solid state fermentation of several treatments of whole or chopped larvae combined with barley meal. One treatment also included lactic acid bacteria (not further specified). In this study, pH values around 4.5 were reached. To the best of our knowledge, our study is the first to report on the fermentation of (almost) pure BSFL with the use of an established starter culture. In our study, we pulverised the larvae to obtain a paste (i.e., more intensive fragmentation than gently crushing) because our earlier work with yellow mealworms had shown that whole larvae did not yield a good fermentation process. In a paper by Borremans et al. [25], we described that fermentation of whole larvae caused a ‘rotten egg’ odour, likely because the starter culture was not able to penetrate through the exoskeleton reaching the inside mass to ferment it. Based on the report of Mogodiniyai Kasmaei et al. [34] and our own experience with mealworms, we opted for a thorough fragmentation to obtain a paste.

The pH values of the pulverised BSFL obtained before incubation are values that allow growth of a wide variety of micro-organisms. As this was the first time to ferment pulverised larvae, the ideal fermentation time was not known and incubation was performed during seven days. The on-line pH measurements demonstrate that the use of a starter culture leads to a faster and more certain pH reduction than when no starter is used. The pH reduction accomplished with Bactoferm^®^ F-LC ended after about 24 h, but *L. farciminis* leads to a lower end pH when incubation is longer than 24 h. Nevertheless, the pH reduction accomplished by the starters in the BSFL paste was much lower than in the mealworm paste in earlier research [28]: the pH of unfermented mealworm paste was 6.54 ± 0.02, of paste fermented seven days with Bactoferm^®^ F-LC 4.50 ± 0.01 and of paste fermented seven days with *L. farciminis* 4.39 ± 0.04). It is not exactly known why a pH reduction seems easier to establish in mealworm than in BSFL paste, probably the composition of BSFL has a larger buffering capacity than that of yellow mealworms. As described by Mennah-Govela et al. [36], the buffering capacity of a food (or feed) matrix involves the ability of the material to resist changes in pH after addition of acid (in this case organic acids produced by the starter cultures during fermentation) or alkali. Not all factors contributing buffering capacity are clearly understood yet, but the chemical composition of the matrix, and in particular the presence of acid/base groups, interacting in a different way with free protons in the matrix, play a role. The buffering capacity of BSFL or other edible insects is not investigated yet, but it can be assumed that differences in their composition contribute to an explanation for a difference in buffering capacity.

A particular point of attention that came up from the microbiological counts is the presence of bacterial spores. It is known from earlier research that BSFL can contain high amounts of endospores, as similar values were also found in BSFL by Wynants et al. [37]. Blanching is known not to reduce endospore counts in yellow mealworms [12]. The most important bacterial genera that form endospores are *Clostridium* and *Bacillus*, and these genera also contain certain species that are food pathogens and/or spoilage organisms. The presence of bacterial endospores in feed or food is not a food safety problem, as long as the spores do not germinate. Spore germination can be prevented in general by a low pH of the matrix, for instance achieved by fermentation. The pH of the matrix should be below the value necessary for spore germination, which varies for different spore-forming species. For instance, Foerster [38] reported that the optimum pH for germination of spores of some thermophilic *Bacillus* species was found to be 6.00. Ishida et al. [39] showed that the optimum pH for spore germination of *Bacillus subtilis* ranged between 7.4 and 5.4 depending on the temperature. Valero et al. [40] investigated a *Clostridium sporogenes* cocktail and concluded that normally, it does not germinate at a pH below 5.0. In contrast, Wong et al. [41] demonstrated that *Clostridium botulinum* spores can germinate even below pH 4.6. In the context of fermented foods, Hutkins [42] stated that a pH reduction to a value below 5.10 is necessary to provide a barrier against most foodborne pathogens. In our experiments, this value was never reached, neither in the samples where no starter was added nor in the samples with starter. Based on this study, we can conclude that (aerobic) endospores pose a potential danger in fermented BSFL paste. This is in contrast to fermented yellow mealworm paste, where the amount of aerobic endospores is typically lower [25] and where, on top of that, pH is lower after a fermentation process as applied in [25].

For fermentation, it can be concluded from our study that it is applicable as a technology, but that spores should receive special attention. Fermentation can exist next to heat treatment as a preservation technology. Future research on this topic may elucidate whether fermentation can provide an additional advantage over heat treatment, by a better preservation of nutrients present in the insects.

To the authors’ knowledge, vacuum packaging has not yet been investigated as a preservation technology for insects, and our results cannot be compared to findings of others. Based on our data, the technology does not seem to be able to replace cooling or to bring an additional advantage on top of cooling. This is the case for both living and killed larvae, either by blanching or freezing. Further research is necessary to find the most optimal (in terms of obtained shelf life in combination with capital and operational expenditures) technologies for BSFL and other insects. In the case of killed insects, our study demonstrated that the killing method can affect the microbiological condition at the start of the storage period. Likely also the chemical composition may be impacted. Hence, when designing storage methods, the killing technique should be taken into account. MAP encompasses vacuum packaging, but also packaging in altered gas compositions. As experienced in other research (data not yet published), killed BSFL are prone to oxidation, even when kept frozen. Packaging in 100% N_2_ may prevent or limit oxidation processes, but it remains to be investigated in future research.

## Figures and Tables

**Table 1:** Results of off-line pH measurements before incubation, after incubation and after cooled storage of control Scheme 3 ± standard deviation. ^a^ Means for control and starter treatment per starter tested and per measuring time point do not differ significantly when they have the same superscript (small letter). ^A,B^ Means for each individual treatment and for the three time points do not differ significantly when they have the same superscript (capital letter).

Starter Tested	Treatment	pH		
		Before Incubation	After Incubation	After Storage
Bactoferm^®^ F-LC	No starter (control)	7.06 ± 0.37 ^A,a^	6.18 ± 0.83 ^B,a^	5.96 ± 0.16 ^B,a^
	Starter	7.12 ± 0.38 ^A,a^	6.09 ± 0.92 ^B,a^	6.43 ± 0.83 ^A,B,a^
*L. farciminis*	No starter (control)	7.00 ± 0.24 ^A,a^	6.99 ± 1.34 ^A,a^	6.93 ± 1.13 ^A,a^
	Starter	7.19 ± 0.09 ^A,a^	6.40 ± 1.07 ^A,B,a^	6.23 ± 0.60 ^B,a^

**Table 2:** Microbial counts before and after 6 days of storage without applying vacuum (control) or in vacuum, at room temperature (RT) or at 4 °C and for larvae that were killed by either blanching or freezing. Results originate from one replicate.

Killing Method	Storage Temperature	Packaging Condition	Microbial Counts (log cfu/g)			
			Total Aerobic Viable Counts	Entero- Bacteriaceae	Lactic Acid Bacteria	Aerobic Bacterial Endospores
Blanching		Before packaging	4.75	<1.00 ^†^	2.33	3.97
	RT	Control	9.05	8.40	7.63	6.00
		Vacuum	8.85	8.45	6.89	5.26
	4 °C	Control	<4.97 ^†^	<4.97 ^†^	<3.97 ^†^	3.25
		Vacuum	<4.95 ^†^	<4.95 ^†^	<3.95 ^†^	3.09
Freezing		Before packaging	8.28	6.37	7.42	4.83
	RT	Control	9.56	8.28	8.13	3.66
		Vacuum	9.16	8.24	7.74	4.73
	4 °C	Control	7.23	6.35	5.23	4.38
		Vacuum	7.18	7.05	<5.00 ^†^	4.46

^†^ Results preceded by “<” indicate the detection limit for that sample and imply that the actual value is lower than that detection limit. Detection limits can differ for samples, depending on the serial dilutions that were plated for the samples.

## Data Availability

The data presented in this study are available in the Supplementary Materials S1 and S2 or available on request from D.V. or L.V.C.

## Supplementary Materials

The following are available on-line at https://www.mdpi.com/article/10.3390/insects12080714/s1, Figure S1: Oxygen and carbon dioxide concentration during storage at room temperature or at 4 °C of packages of larvae killed by blanching. Packages were only sealed (no vacuum = control) or were vacuum packaged prior to storage. Data points are the mean of one or two packages. Figure S2: Oxygen and carbon dioxide concentration during storage at room temperature or at 4 °C of packages of larvae killed by freezing. Packages were only sealed (no vacuum = control) or were vacuum packaged prior to storage. Data points are the mean of one or two packages.
