# Supplementary figures and images for: Potential of Fermentation and Vacuum Packaging Followed by Chilling to Preserve Black Soldier Fly Larvae (Hermetia illucens)
Source: Insects. 2021 Aug 10;12(8):714. doi: 10.3390/insects12080714 (PMC8396865; doi:10.3390/insects12080714)

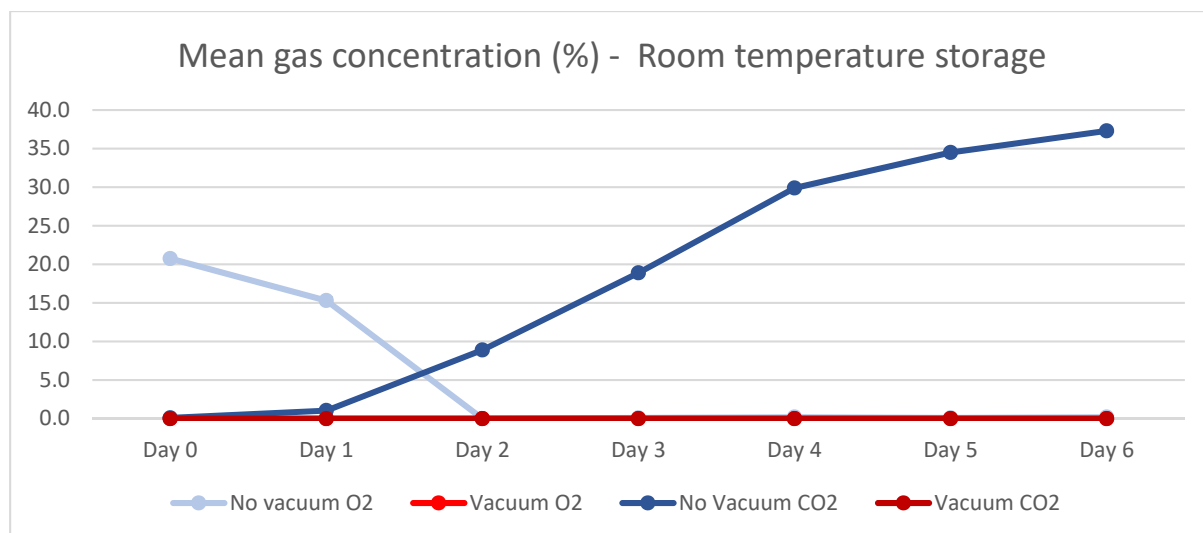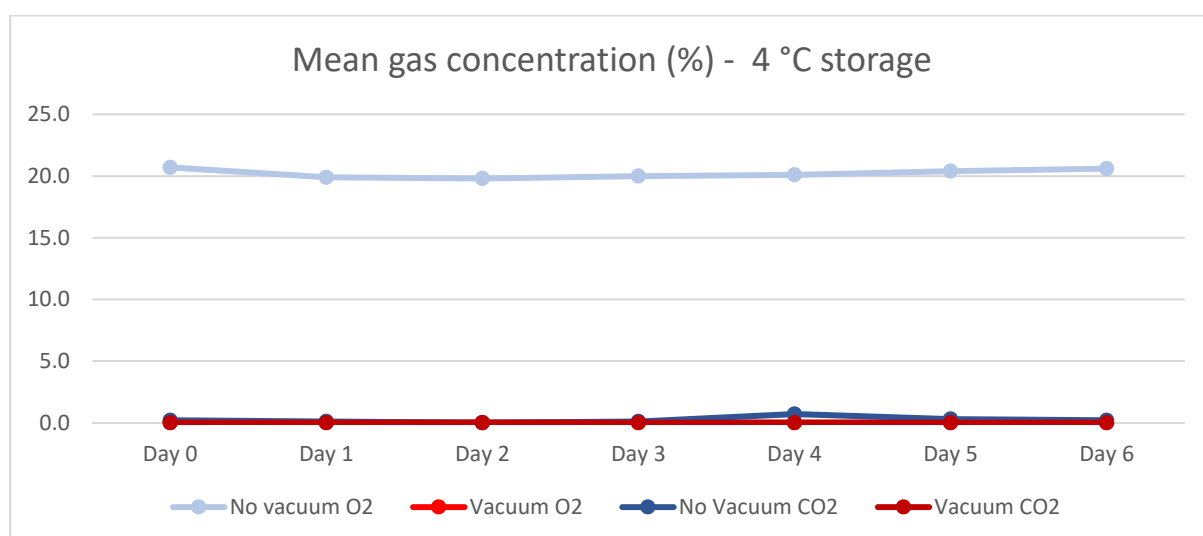

Supplement: Supplementary file 1 [file insects-12-00714-s001.zip › Figure S1 - Gas composition blanched larvae.pdf]

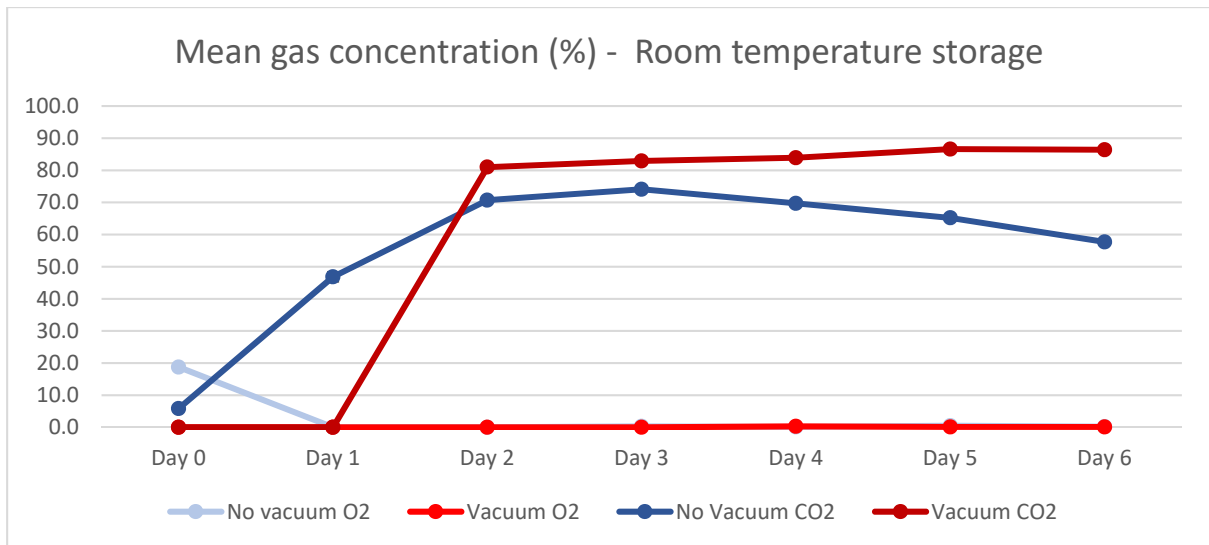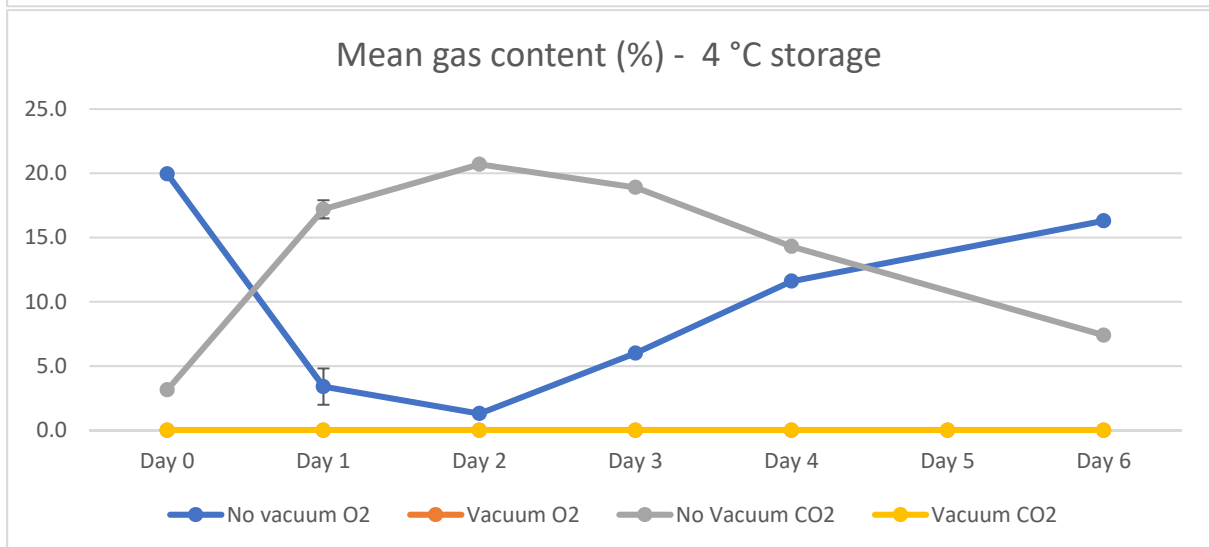

Supplement: Supplementary file 1 [file insects-12-00714-s001.zip › Figure S2 - Gas composition frozen larvae.pdf]
